# Pathologic response and safety to neoadjuvant PD-1 inhibitors and chemotherapy in resectable squamous non-small-cell Lung cancer

**DOI:** 10.3389/fonc.2022.956755

**Published:** 2022-10-14

**Authors:** Liang Shi, Qiyi Meng, Li Tong, Hongxia Li, Yujie Dong, Chongyu Su, Zhe Liu

**Affiliations:** ^1^ Department of Medical Oncology, Beijing Chest Hospital, Beijing Tuberculosis and Thoracic Tumor Research Institute, Capital Medical University, Beijing, China; ^2^ Department of Pathology, Beijing Chest Hospital, Beijing Tuberculosis and Thoracic Tumor Research Institute, Capital Medical University, Beijing, China; ^3^ Department of Thoracic Surgery, Beijing Chest Hospital, Beijing Tuberculosis and Thoracic Tumor Research Institute, Capital Medical University, Beijing, China

**Keywords:** resectable non-small-cell lung cancer, squamous cell carcinoma, neoadjuvant chemoimmunotherapy, programmed death-1 inhibitors, pathologic response

## Abstract

**Background:**

Several randomized studies have shown that the combination of programmed cell death 1 (PD-1) inhibitor and chemotherapy is efficacious as a treatment for advanced non-small-cell lung cancer (NSCLC). However, in the neoadjuvant setting, there is scarce evidence of the effectiveness and safety of the combinations in squamous NSCLC. We conducted a retrospective study to evaluate neoadjuvant PD-1 inhibitor plus chemotherapy in resectable squamous NSCLC.

**Methods:**

Patients from Beijing Chest Hospital, Capital Medical University, between October 2019 and October 2021, treated with PD-1 inhibitors and chemotherapy for resectable squamous NSCLC were retrospectively studied. The primary objectives were to assess the pathological tumor response and safety of neoadjuvant PD-1 inhibitors and chemotherapy.

**Results:**

63 patients with resectable squamous NSCLC stage IIA-IIIB were included. Two to four cycles of PD-1 inhibitors (37 cases with camrelizumab, 11 cases with toripalimab, 8 cases with tislelizumab, and 7 cases with sintilimab) and chemotherapy were administered prior to surgery. 42 patients (66.7%) achieved a major pathologic response (MPR), including 25 (39.7%) with a pathologic complete response (pCR). Twenty-one patients (33.3%) experienced grade 3 neoadjuvant treatment-related adverse events (TRAEs), and no patient had grade 4 or 5 TRAE.

**Conclusion:**

Neoadjuvant PD-1 inhibitors and chemotherapy are feasible therapies for resectable squamous NSCLC. It was associated with a 66.7% MPR rate, 39.7% pCR rate, and tolerable toxicity.

## Introduction

In non-small-cell lung cancer (NSCLC), squamous NSCLC (sqNSCLC) represents approximately 25% to 30% (1), and it is associated with a shorter survival time than nonsquamous NSCLC (2, 3). Squamous NSCLC has historically been treated almost exclusively with cytotoxic chemotherapy due to the lack of targetable aberrations (4).

Patients with resectable NSCLC at high recurrence risk may benefit from neoadjuvant or adjuvant chemotherapy; however, the 5-year overall survival (OS) gain is only 5% (5, 6). Inhibitors of programmed death receptor 1 (PD-1) and its ligand programmed death‐ligand 1 (PD-L1) are effective in the treatment of advanced squamous and nonsquamous NSCLC (7–11). These PD-1/PD-L1 inhibitors are evaluated in multiple clinical trials, rapidly moving from advanced NSCLC to resectable stages and from palliative to curative strategies.

Single-arm phase 2 studies with immunotherapy agents as monotherapy or in combination have recently shown encouraging outcomes (pathologic complete response, event-free survival, and OS) in the neoadjuvant setting (12–16). CheckMate 816 is a randomized, phase 3, open-label study evaluating nivolumab-plus-chemotherapy versus chemotherapy as neoadjuvant treatment for resectable NSCLC, The CheckMate 816 showed statistically significant improvements in the primary endpoints of event-free survival (EFS, median EFS was 31.6 months in the nivolumab-plus-chemotherapy arm and 20.8 months in the chemotherapy-alone arm; hazard ratio, 0.63; 97.38% CI, 0.43 to 0.91), and the pathologic complete response (pCR, pCR rate was 24% in the nivolumab-plus-chemotherapy arm and 2.2% in the chemotherapy-alone arm, odds ratio, 13.94; 99% CI, 3.49 to 55.75) (17). As a result of CheckMate 816, the FDA approved using nivolumab in combination with platinum-doublet chemotherapy for resectable NSCLC patients in the neoadjuvant setting, but in China, this strategy has not yet been approved.

However, in neoadjuvant therapy for NSCLC, few clinical studies on neoadjuvant treatment are designed for squamous cell carcinoma. Therefore, there is scarce evidence of the effectiveness and safety of the neoadjuvant chemoimmunotherapy in squamous NSCLC, especially in several ones approved in China for first-line treatment in advanced sqNSCLC (18–20). In addition, the pathological response of neoadjuvant chemoimmunotherapy in squamous cell carcinoma is not clear. Therefore, we conducted a retrospective study to evaluate pathological response and safety of neoadjuvant PD-1 inhibitor plus chemotherapy in resectable squamous NSCLC.

## Materials and methods

### Patient population

**Figure 1:**
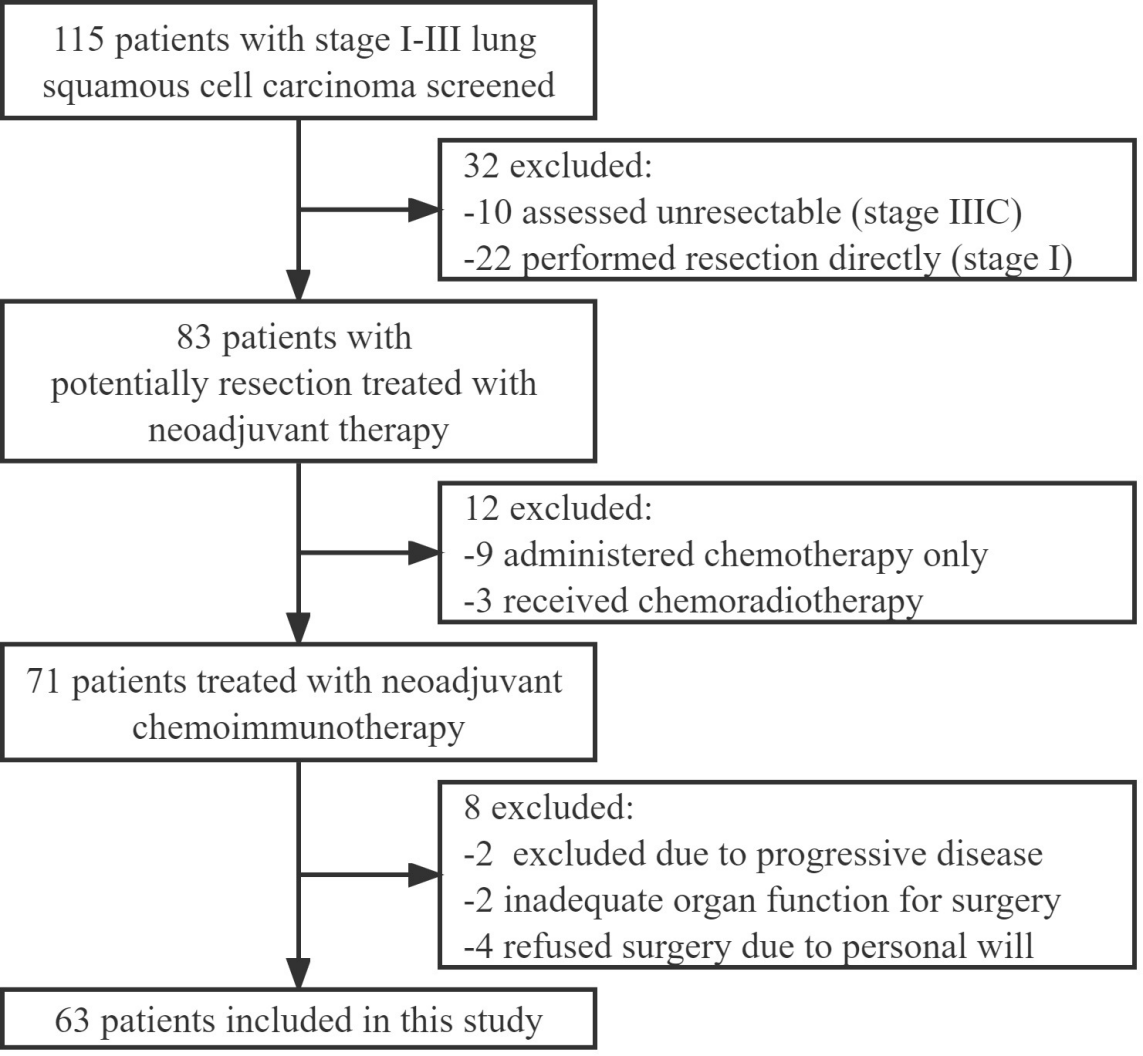
Flow diagram of patients included in this study.

Patients from Beijing Chest Hospital, Capital Medical University, treated with PD-1 inhibitors and chemotherapy for resectable sqNSCLC between October 2019 and October 2021, were retrospectively studied. The inclusion criteria were as follows: age 18 years or older, confirmed histological diagnosis of sqNSCLC, Eastern Cooperative Oncology Group performance status (ECOG PS) ≤ 2, clinical stage IIA-IIIB before the treatment, and ≥2 neoadjuvant treatment cycles, adequate organ function and undergone surgical resection. The exclusion criteria were as follows: had previous treatment before diagnosis or lacked completed radiological or pathological data. The resectable criteria were followed by defining the resectability status of the National Comprehensive Cancer Network (NCCN) Guidelines for all stage IIA-IIIA cases. In terms of stage IIIB patients, the cases including tumor T3/T4 with single-station non-bulky N2 disease of mediastinal lymph nodes, excluding tumor T3/T4 with multi-station N2 disease or bulky N2 disease, were judged as potentially resectable or marginal resectable. Therefore, only cases that met the resectable criteria were administrated for neoadjuvant chemoimmunotherapy. Finally, 63 patients were included in the study (**Figure 1**). The study was carried out following the Declaration of Helsinki (as revised in 2013). It was reviewed and approved by the institutional review board (IRB)/ethics committee of Beijing Chest Hospital, Capital Medical University. In the neoadjuvant chemoimmunotherapy for operable NSCLC of this study, all PD-1 inhibitors were given for off-label use. All patients were fully informed and signed informed consent before starting treatment.

The collected clinicopathologic data of the patients included sex, age, smoking history, ECOG PS, PD-L1 expression (22C3 PD-L1 antibody, Dako, Denmark), clinical TNM (cTNM) stage, neoadjuvant treatment regimen, treatment cycle, surgical treatment, radiological and pathological efficacy evaluation, and treatment-related adverse events (TRAEs). In addition, clinical TNM was determined according to the 8th edition of the lung cancer staging system of the American Joint Committee on Cancer (21).

### Treatment regimen and surgery

All of the included patients were scheduled to receive surgery within 4-6 weeks after neoadjuvant chemoimmunotherapy that consisted of 2-4 cycles of a conventional platinum-based doublet chemotherapy regimen with PD-1 inhibitor on day 1 of each 21-day cycle. Patients received one of the following PD-1 inhibitors intravenously as neoadjuvant immunotherapy: camrelizumab (200 mg), toripalimab (240 mg), tislelizumab (200 mg), or sintilimab (200 mg).

As per standard institutional procedures, all surgical resections were performed with thoracotomy or video-assisted thoracoscopic surgery.

### Treatment evaluation

The primary objectives were to assess the pathological tumor response of neoadjuvant PD-1 inhibitors and chemotherapy. The pathological tumor response endpoints were MPR, defined as ≤10% residual viable tumor cells in the primary tumor and sampled lymph nodes, and pCR, defined as the complete absence of residual viable tumor cells in the primary tumor and sampled lymph nodes (22).

Secondary endpoints were the imaging response and safety profile of the combination.

Contrast-enhanced CT scans were repeated to assess objective imaging response within seven days before surgery. The imaging responses were evaluated for all patients per the Response Evaluation Criteria in Solid Tumors (RECIST) version 1.1 (23), and the therapeutic response was considered as complete response (CR), partial response (PR), stable disease (SD), or progression disease (PD). The safety endpoints included treatment-related adverse events according to the Common Terminology Criteria for Adverse Events (CTCAE, v.5.0).

### Statistical analysis

All statistical analyses were performed with Stata version 17.0 (StataCorp, TX, USA) or GraphPad Prism version 9.0 (GraphPad Software Inc., CA, USA). Frequency tabulation and summary statistics for the patient’s baseline characteristics, surgical outcomes, and safety evaluation provided data distribution characteristics. Continuous variables were expressed as medians with ranges. Categorical variables were expressed as numbers with percentages. The association of baseline characteristics and pathological response were conducted with the Fisher’s exact test. The association between the clinical response and the pathological response was performed with Pearson correlation coefficient analysis. A two-sided p value less than 0.05 was considered statistically significant.

## Results

### Patient characteristics

Sixty-three patients with resectable squamous NSCLC stage IIA-IIIB were included (**Table 1**). Of these patients, eight were females and 55 males aged from 47 to 75 years old (median age of 63 years old). Most patients (73.0%) had stage IIIA to IIIB disease, according to the IASLC eighth edition of the TNM Classification for Lung Cancer. PD-L1 expression before treatment was detected by the PD-L1 IHC 22C3 pharmDx assay. For the 40 patients with available PD-L1 data, 32 patients (50.8%) had a PD-L1 tumor proportion score of 1% or higher.

**Table 1:** Clinicopathologic characteristics of 63 patients.

Characteristic	Value or No. of Patients	%
Patients	63	
Age, years		
Median	63	
Range	47-75	
Sex		
Female	8	12.7%
Male	55	87.3%
Smoking status		
Never	17	27.0%
Current/Former	46	73.0%
ECOG PS		
0	39	61.9%
1	24	38.1%
Clinical T stage		
T1	3	4.8%
T2	20	31.7%
T3	22	34.9%
T4	18	28.6%
Clinical N stage		
N0	18	28.6%
N1	17	27.0%
N2	28	44.4%
Clinical stage (8th edition)		
IIA	3	4.8%
IIB	14	22.2%
IIIA	31	49.2%
IIIB	15	23.8%
PD-L1 expression		
Positive (≥1%)	32	50.8%
≥1%-49%	13	20.6%
≥50%	19	30.2%
Negative (<1%)	8	12.7%
NA	23	36.5%
Neoadjuvant PD-1 inhibitor regimen		
Camrelizumab	37	58.7%
Toripalimab	11	17.5%
Tislelizumab	8	12.7%
Sintilimab	7	11.1%
Neoadjuvant treatment cycles		
2	41	65.1%
3	18	28.6%
4	4	6.3%

ECOG PS, Eastern Cooperative Oncology Group performance score; NA, not applicable; PD-1, programmed death 1; PD‐L1, programmed death‐ligand 1.

### Neoadjuvant treatment and imaging efficacy

Two to four cycles of PD-1 inhibitors (37 cases with camrelizumab, 11 cases with toripalimab, 8 cases with tislelizumab, and 7 cases with sintilimab) and chemotherapy were administered prior to surgery (**Table 1**). The clinical activity of the chemoimmunotherapy neoadjuvant combination was evaluated according to the RECIST v.1.1 criteria. In particular, 43 out of the 63 cases achieved a partial response (PR, 68.3%), while 20 patients presented a stable disease (SD, 31.7%).

### Surgical treatment and pathological efficacy

All 63 patients received R0 surgical resection. The results for surgical treatment are shown in **Table 2**. Surgical methods included video-assisted thoracoscopic surgery (VATS) (n=32) and thoracotomy (n=31), including 47 (74.6%) lobectomy, 9 (14.3%) bilobectomy and 7 (11.1%) pneumonectomy. The median days of hospitalization after surgery operations was 10 (range, 1–68), the median operation time was 154 (range, 85–310) minutes, and the median amount of estimated blood loss was 150 mL (50–1100 mL). One patient died within 48 hours of lobectomy. He had a clinical T3N2 primary tumor. Radiographic SD was observed after two cycles of neoadjuvant chemoimmunotherapy, which resulted in a technically challenging resection. The patient developed severe hypoxemia, required ventilator support, and died 48 hours postoperatively.

**Table 2:** Surgical outcomes.

Surgical outcomes	Patients (n=63)
Operation time (minutes)	
Median	154
Range	85-310
Hospitalization after surgery (days)	
Median	10
Range	1-68
Estimated blood loss (mL)	
Median	150
Range	50-1100
R0 resection, n (%)	
Yes	63 (100%)
No	0 (0%)
Extent of resection, n (%)	
Lobectomy	47 (74.6%)
Bilobectomy	9 (14.3%)
Pneumonectomy	7 (11.1%)
Surgical methods, n (%)	
Video-assisted thoracoscopic surgery	32 (50.8%)
thoracotomy	31 (49.2%)
Perioperative death, n (%)	1 (1.6%)

In total, 42 patients (66.7%) achieved a major pathologic response (MPR), including 25 (39.7%) with a pathologic complete response (pCR) in the primary tumor and sampled lymph nodes. In two patients, the primary tumor disappeared, but the regional lymph node involvement persisted, achieving an MPR in the final overall evaluation.

**Figure 2:**
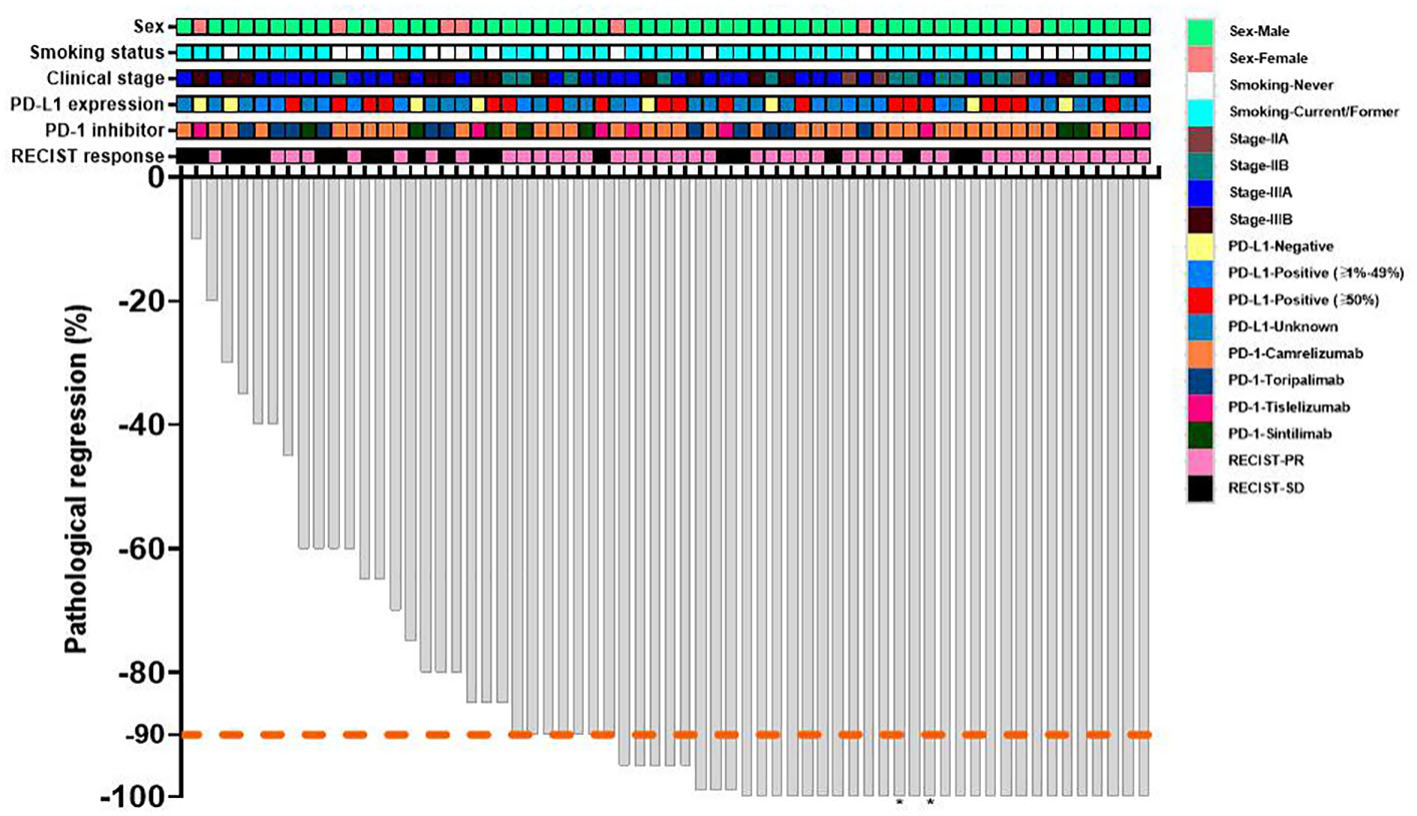
Waterfall plot of pathological response of neoadjuvant therapy. Each bar represents one patient. The upper rows show clinical characteristics and radiological responses. PD-L1, programmed death-ligand 1; PD-1, programmed death 1; RECIST, response evaluation criteria in solid tumors; PR, partial response; SD, stable disease. *The regional lymph node of dissection involvement persisted.

**Figure 3:**
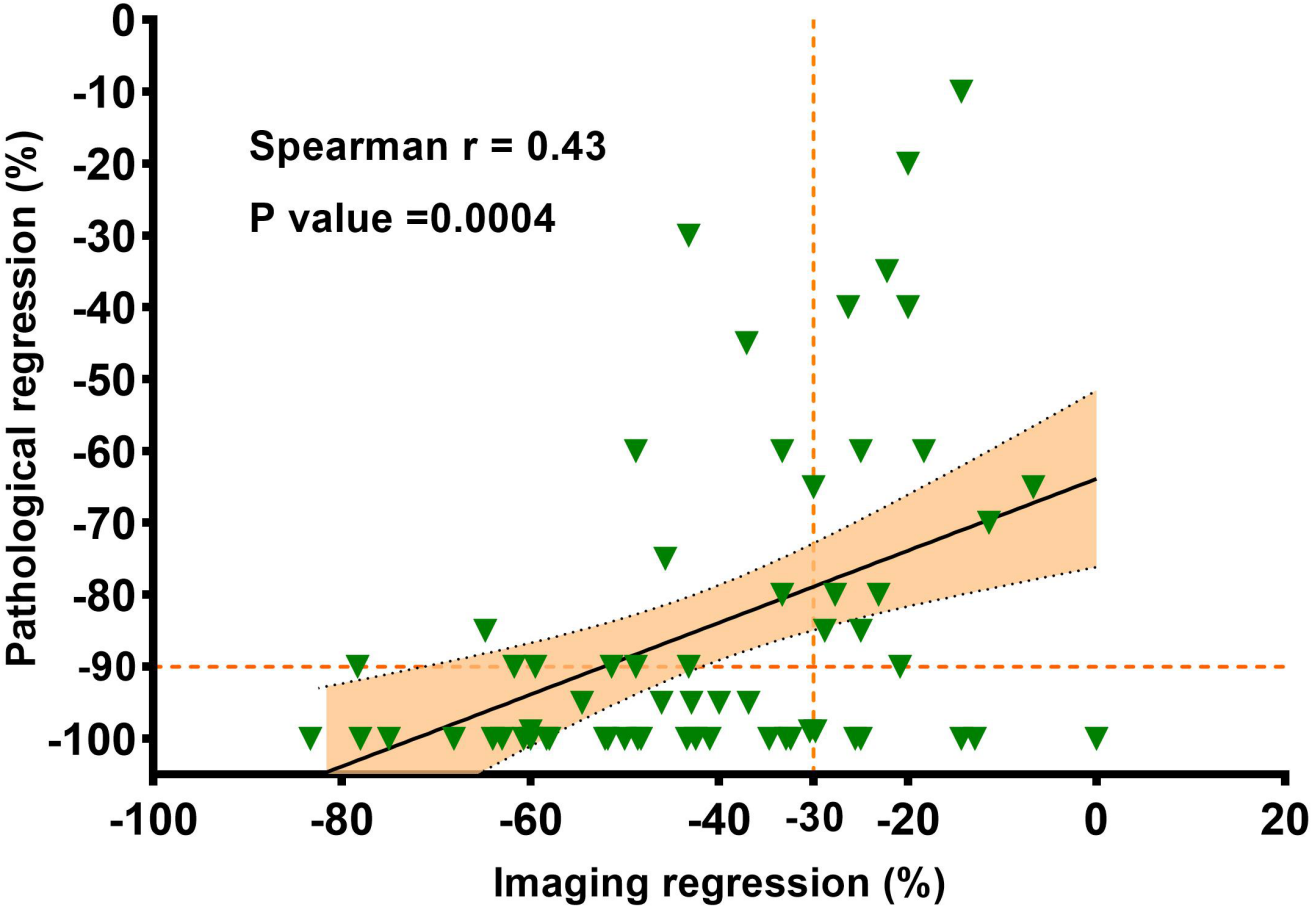
The correlation between the imaging regression and the pathologic regression. Pearson correlation coefficient and two-sided P value are shown.

**Figure 4:**
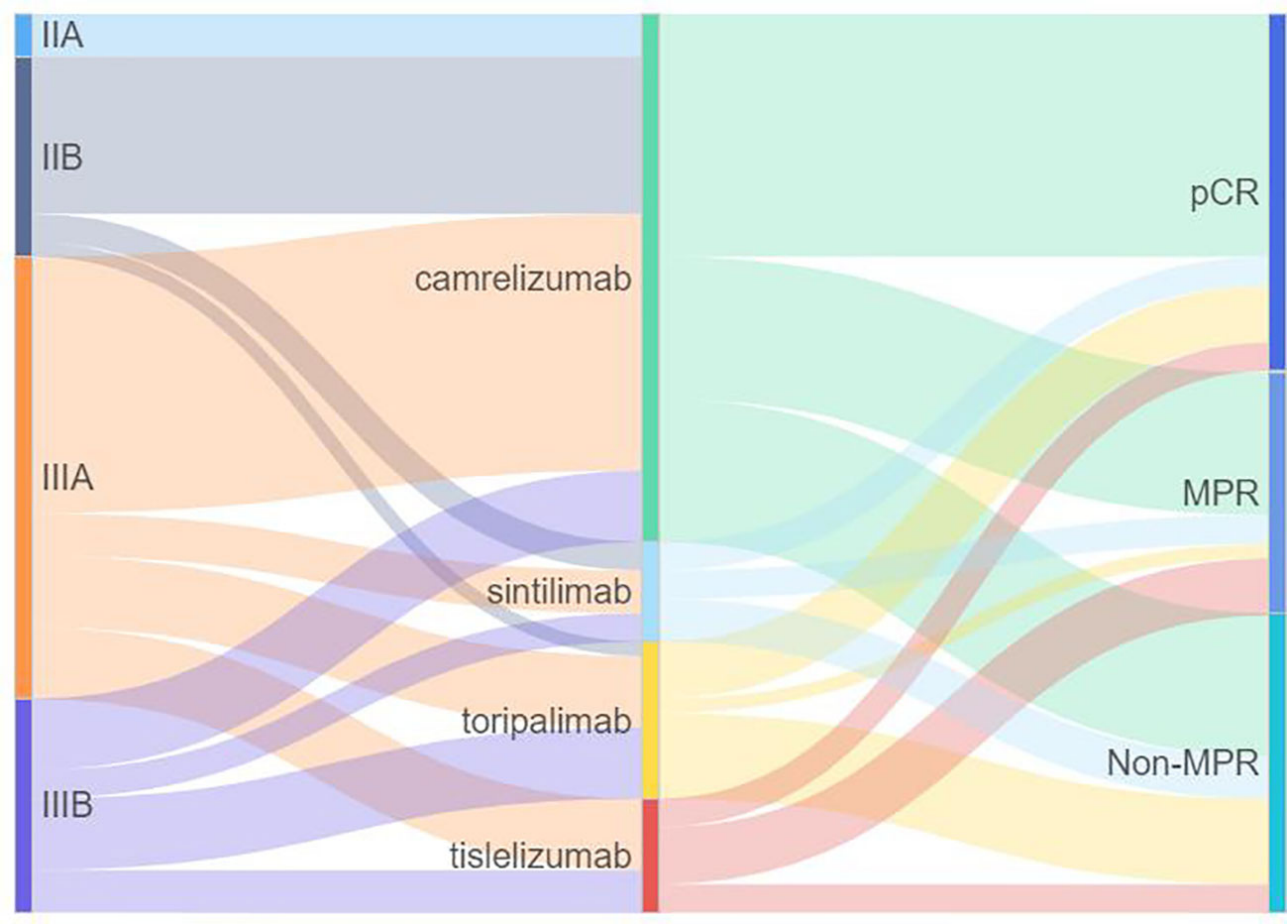
Sankey diagram of the relationship between pathological response of neoadjuvant therapy in different clinical stages. PD-1, programmed death 1; MPR, major pathologic response; pCR, pathologic complete response.

The waterfall plot shows pathological regression in the resected primary lung tumor after neoadjuvant administration, according to the subgroup of sex, smoking status, clinical TNM stage, PD-L1 expression, PD-1 inhibitor regimen, and RECIST response (**Figure 2**). There was correlation between the imaging regression and pathological regression (Spearman correlation coefficient = 0.43; P = 0.0004; **Figure 3**). The MPR was related to the clinical lymph nodal stage (Fisher’s exact test P = 0.009) and clinical TNM stage (Fisher’s exact test P = 0.027). The pCR was only related to the clinical TNM stage (Fisher’s exact test P = 0.047, **Table 3**). The Sankey diagram shows the degree of relationship between the pathological response of neoadjuvant therapy in different clinical stages (**Figure 4**).

**Table 3:** Association between clinical characteristics and pathological response.

Characteristic	MPR/pCR	Non-MPR	P Value	pCR	Non-pCR	P Value
		(n=42)		(n=21)			(n=25)	(n=38)	
Age, years						
<65	21 (50%)	14 (67%)	0.21	13 (52%)	22 (58%)	0.65
≥65	21 (50%)	7 (33%)		12 (48%)	16 (42%)	
Sex						
Female	3 ( 7%)	5 (24%)	0.061	2 ( 8%)	6 (16%)	0.36
Male	39 (93%)	16 (76%)		23 (92%)	32 (84%)	
ECOG PS						
0	9 (21%)	8 (38%)	0.16	6 (24%)	11 (29%)	0.67
1	33 (79%)	13 (62%)		19 (76%)	27 (71%)	
Smoking status						
Never	28 (67%)	11 (52%)	0.27	18 (72%)	21 (55%)	0.18
Current/Former	14 (33%)	10 (48%)		7 (28%)	17 (45%)	
Clinical T stage						
T1	3 ( 7%)	0 ( 0%)	0.78	3 (12%)	0 ( 0%)	0.22
T2	13 (31%)	7 (33%)		8 (32%)	12 (32%)	
T3	15 (36%)	7 (33%)		8 (32%)	14 (37%)	
T4	11 (26%)	7 (33%)		6 (24%)	12 (32%)	
Clinical N stage						
N0	14 (33%)	4 (19%)	0.009	10 (40%)	8 (21%)	0.095
N1	15 (36%)	2 (10%)		8 (32%)	9 (24%)	
N2	13 (31%)	15 (71%)		7 (28%)	21 (55%)	
Clinical stage (8th edition)						
IIA	3 ( 7%)	0 ( 0%)	0.027	3 (12%)	0 ( 0%)	0.047
IIB	13 (31%)	1 ( 5%)		8 (32%)	6 (16%)	
IIIA	19 (45%)	12 (57%)		10 (40%)	21 (55%)	
IIIB	7 (17%)	8 (38%)		4 (16%)	11 (29%)	
PD-L1 expression						
Negative (<1%)	4 (10%)	4 (19%)	0.65	3 (12%)	5 (13%)	0.84
Positive (≥1%-49%)	8 (19%)	5 (24%)		6 (24%)	7 (18%)	
Positive (≥50%)	14 (33%)	5 (24%)		6 (24%)	13 (34%)	
NA	16 (38%)	7 (33%)		10 (40%)	13 (34%)	
Neoadjuvant PD-1 inhibitor regimen						
Camrelizumab	27 (64%)	10 (48%)	0.34	17 (68%)	20 (53%)	0.69
Toripalimab	5 (12%)	6 (29%)		4 (16%)	7 (18%)	
Tislelizumab	6 (14%)	2 (10%)		2 ( 8%)	6 (16%)	
Sintilimab	4 (10%)	3 (14%)		2 ( 8%)	5 (13%)	
Neoadjuvant treatment cycles						
2	29 (69%)	12 (57%)	0.61	15 (60%)	26 (68%)	0.57
3	11 (26%)	7 (33%)		9 (36%)	9 (24%)	
4	2 ( 5%)	2 (10%)		1 ( 4%)	3 ( 8%)	

MPR, major pathologic response; pCR, pathologic complete response; ECOG PS, Eastern Cooperative Oncology Group performance score; NA, not applicable; PD-1, programmed death 1; PD‐L1, programmed death‐ligand 1.

### Safety

Treatment-related adverse events were reported for 62 (98.4%) patients treated with neoadjuvant PD-1 inhibitors plus chemotherapy. Most of the adverse events were in grades 1-2. Grade 3 treatment-related adverse events occurred in 21 (33.3%) patients, including decreased neutrophil count 11 (17.5%) patients, pneumonia 7 (11.1%), and decreased white blood cell count 5 (7.9%), (**Table 4**). No grade 4-5 toxicities occurred during the neoadjuvant treatment phase.

**Table 4:** Treatment-related adverse events during neoadjuvant treatment (n=63).

	Any grade, n (%)	Grade 1-2, n (%)	Grade 3, n (%)
Any treatment-related adverse event	62 (98.4)	62 (98.4)	21 (33.3)
**Hematological toxicities**			
Anemia	45 (71.4)	45 (71.4)	0
Neutrophil count decreased	30 (47.6)	19 (30.2)	11 (17.5)
White blood cell decreased	20 (31.7)	15 (23.8)	5 (7.9)
Platelet count decreased	7 (11.1)	7 (11.1)	0
**Nonhematological toxicities**			
ALT/AST increased	26 (41.3)	26 (41.3)	0
Vomiting	23 (36.5)	23 (36.5)	0
Hypoalbuminemia	22 (34.9)	22 (34.9)	0
Blood bilirubin increased	16 (25.4)	16 (25.4)	0
TSH increased	13 (20.6)	13 (20.6)	0
Pneumonia	12 (19.0)	5 (7.9)	7 (11.1)
Hyponatremia	11 (17.5)	11 (17.5)	0
Serum amylase increased	10 (15.9)	10 (15.9)	0
Hyperuricemia	9 (14.3)	9 (14.3)	0
Constipation	8 (12.7)	8 (12.7)	0
Rash	7 (11.1)	7 (11.1)	0
Hypokalemia	6 (9.5)	6 (9.5)	0
Creatinine increased	6 (9.5)	6 (9.5)	0
Anorexia	5 (7.9)	5 (7.9)	0
Fatigue	3 (4.8)	3 (4.8)	0
Alopecia	2 (3.2)	2 (3.2)	0
Diarrhea	2 (3.2)	2 (3.2)	0

ALT, alanine aminotransferase; AST, aspartate aminotransferase; TSH, thyroid stimulating hormone.

## Discussion

Resectable sqNSCLC is usually treated with a combination of surgery, radiation, and systemic chemotherapy. However, it has been proven that immunotherapy is a very effective front-line treatment for advanced sqNSCLC (8, 11, 18–20). Additionally, perioperative immunotherapy has been proven successful in NSCLC (13, 17, 24), but the effect of chemoimmunotherapy in resectable sqNSCLC has rarely been reported. In this study, we retrospectively analyzed 63 squamous NSCLC with stage II-IIIB treated with neoadjuvant chemoimmunotherapy. Our study revealed that PD-1 inhibitors plus chemotherapy were prescribed preoperatively, thus resulting in 66.7% (42/63) of patients achieving an MPR and 39.7% (25/63) cases achieving a pCR. Meanwhile, no unexpected adverse reactions were observed.

NSCLC is classified into squamous cell carcinomas and non-squamous cell carcinomas based on their unique biological behavior, clinical molecular characteristics, and therapeutic responses (25). The study found that compared with adenocarcinoma, the expression of PD-L1 in squamous cell carcinoma is more common, and the infiltration of macrophages and other immune cells is more prominent, which brings an opportunity for the treatment of patients with advanced squamous cell carcinoma, and also leads to the different response of squamous cell carcinoma and nonsquamous cell carcinoma to immunotherapy (26).

Notably, our study only included patients with squamous cell carcinoma. After neoadjuvant immunotherapy combined with chemotherapy, we achieved an excellent pathological response from a numerical point of view. Two-thirds of the patients obtained MPR, and nearly 40% of the cases achieved pCR. Our findings further confirmed the findings of previous small samples of neoadjuvant immunotherapy for lung squamous carcinoma (27, 28).

A major pathological response is more likely to be observed in patients with squamous cell carcinoma (26%) than in those with adenocarcinoma (12%) following neoadjuvant chemotherapy studies, possibly because of greater baseline tumor necrosis in squamous cell carcinomas (29). However, the pCR rates of squamous and nonsquamous NSCLC to neoadjuvant nivolumab plus chemotherapy were similar in the CheckMate 816 study, with 25.3% in squamous and 22.8% in nonsquamous. Therefore, more studies are needed to investigate whether the efficacy of neoadjuvant immunotherapy varies against squamous and nonsquamous NSCLC.

In terms of treatment course before surgery, most previous studies choose 2 to 4 cycles. The neoadjuvant single-agent immunotherapy in CheckMate159 and LCMC3, was performed for two cycles (12, 30). Neoadjuvant immunotherapy combined with chemotherapy in NADIM and CheckMate 816, or a combination of two checkpoint inhibitors in NEOSTAR, was performed for three to four cycles (13, 17, 31). In our study, 41 (65.1%) patients received two cycles of preoperative treatment, 18 (28.6%) patients received three cycles, and only four patients received four cycles of treatment (**Table 1**). In terms of efficacy (**Table 3**), further comparing the difference between 2 cycles treatment and 3-4 cycles treatment, we found no statistical correlation (data not shown). In determining the best neoadjuvant treatment course, various factors are taken into account, including efficacy, timing of surgery, and patient compliance. In order to determine the optimal course of treatment, there is a need for more clinical evidence.

Of the 63 patients included in our study, 43 achieved radiological PR, of which 35 (81.4%) achieved pathological MPR or pCR, we found that there was a positive correlation between the imaging regression and pathological regression (Spearman correlation coefficient = 0.43; P = 0.0004; **Figure 3**). However, 20 patients were evaluated as radiological SD, with seven (35.0%) achieving pathological MPR or pCR. The primary role of immunotherapy promotes the immune cells to infiltrate the tumor and then kill the tumor cells. Patients may benefit from neoadjuvant immunotherapy without initial tumor shrinkage, which is likely to contribute to immune cells infiltrating the tumor (32).

A long-standing method of evaluating neoadjuvant therapy is to examine the pathological changes after surgery. Major pathological response to neoadjuvant treatment is a potential surrogate endpoint for survival (33). Several studies in NSCLC suggest an association between pCR and survival (HR, 0.49; 95% CI, 0.42-0.57) (34). Of note, resectable NSCLC treated with neoadjuvant chemotherapy shows low rates of pCR (median, 4%; range, 0-16%) (33). In CheckMate 816 of neoadjuvant chemoimmunotherapy, the pCR rate was 24% in the nivolumab-plus-chemotherapy arm and 2.2% in the control arm (odds ratio, 13.94; 99% CI, 3.49 to 55.75), the event-free survival appeared to be longer in patients who had a pCR than those who did not (17). Our study found that neoadjuvant PD-1 inhibitors and chemotherapy resulted in a 66.7% MPR rate and a 39.7% pCR rate. Patients who achieved either an MPR or a pCR might benefit long-term survival. In the future follow-up period, this point will be clarified further. For the 40 patients with available PD-L1 data in our study, There was no correlation between the PD-L1 expression of the primary baseline tumor and pathological regression (Spearman correlation coefficient = -0.131; P = 0.42; **Supplementary Figure 1**).

In advanced NSCLC, PD-L1 expression is a critical marker to guide treatment selection. Among patients with PD-L1 expression ≥ 50%, PD-1 or PD-L1 inhibitor monotherapy can be selected for first-line treatment (9, 35, 36), and patients with high PD-L1 expression may benefit more from the combined immunotherapy (18, 37). However, in a chemoimmunotherapy neoadjuvant setting, PD-L1 expression is not an ideal therapeutic or prognostic marker, and the results differ in different studies. A benefit with nivolumab plus chemotherapy was seen across PD-L1 subgroups in CheckMate 816 study, with a greater event-free survival benefit in patients with a tumor PD-L1 expression level of 1% or more than in those with a level of less than 1% (18, 37). There was a significant difference in PD-L1 tumor proportion score between patients who had a complete pathological response and those who had an incomplete pathological response in the NADIM study (p=0.042) (13), but PD-L1 staining was not predictive of survival (38).

The association of PD-L1 expression in tumor tissues with the efficacy and prognosis of neoadjuvant immunotherapy is unclear and requires continued studies with a larger sample size. Neoadjuvant immunotherapy for NSCLC requires biomarkers that accurately predict efficacy to select people who benefit (13, 38). A single biomarker may be challenging to meet the clinical needs of the published clinical studies. Combining multiple biomarkers is the future trend, and the best biomarkers to predict the efficacy also need to be explored.

The limitations of our study include, but are not limited to, the bias of a retrospective single cohort study, the small number of patients who were included, and the lack of survival follow-up. Therefore, larger randomized control studies are needed to reduce bias and determine the most effective PD-1 blockades of neoadjuvant therapy. Furthermore, long-term follow-up of these studies will be necessary to define the role of neoadjuvant PD-1 blockade in reducing recurrences and curing resectable cancers. In addition, PD-L1 was detected in some but not all patients. At the same time, the ctDNA and tumor mutational burden were not recorded in our study, and adequate biomarker studies are needed to identify the best predictive biomarkers of response and to correlate the pathologic response of neoadjuvant chemoimmunotherapy.

Neoadjuvant PD-1 inhibitors and chemotherapy are feasible therapies for resectable squamous NSCLC. It was associated with a 66.7% MPR rate, 39.7% pCR rate, and tolerable toxicity.

## Data availability statement

The raw data supporting the conclusions of this article will be made available by the authors, without undue reservation.

## Ethics statement

This study was approved by the institutional review board (IRB)/ethics committee of Beijing Chest Hospital, Capital Medical University. All patients were fully informed and signed informed consent before starting treatment.

## Author contributions

ZL and LS conceived the study. LS, QM, LT, HL, YD, and CS collected the data. LS, HL, and ZL analyzed the data. LS, QM, LT, HL, YD, CS, and ZL interpreted the data. LS and ZL wrote the first draft of the manuscript. All authors read and contributed to the final version of the manuscript and approved its submission for publication.

## Funding

This study was supported by Beijing Tongzhou District High Level Talent Project (Grant number: YH201910 to ZL and YH201916 to LS).

## Acknowledgments

We thank patients and their families for their support and participation in this study. Part of this work was presented as a poster at 2021 World Conference on Lung Cancer, worldwide virtual event, September 8-14 2021.

## Conflict of interest

The authors declare that the research was conducted in the absence of any commercial or financial relationships that could be construed as a potential conflict of interest.

The reviewer JN declared a shared parent affiliation with the authors to the handling editor at the time of review.

## Publisher’s note

All claims expressed in this article are solely those of the authors and do not necessarily represent those of their affiliated organizations, or those of the publisher, the editors and the reviewers. Any product that may be evaluated in this article, or claim that may be made by its manufacturer, is not guaranteed or endorsed by the publisher.
