# Supplementary material for: Pathologic response and safety to neoadjuvant PD-1 inhibitors and chemotherapy in resectable squamous non-small-cell Lung cancer
Source: Front Oncol. 2022 Oct 14;12:956755. doi: 10.3389/fonc.2022.956755 (PMC9614263; doi:10.3389/fonc.2022.956755)

## Supplementary Figure 1.

PD-L1 expression versus pathologic response (n=40). A: The correlation between the PD-L1 expression and the pathologic regression. Pearson correlation coefficient and two-sided P value are shown. B: PD-L1 expression in different pathologic response.


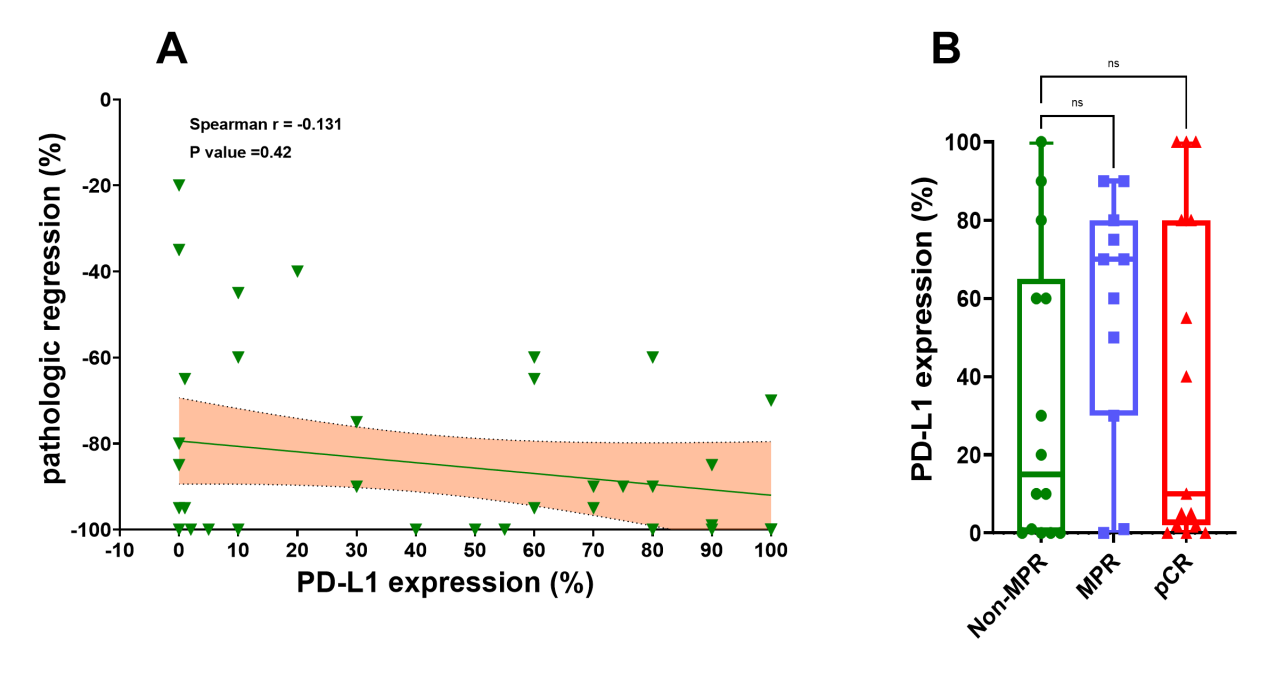

Supplement: Supplementary file 1 [file DataSheet_1.docx]
